# Exploring the Impact of BDNF Val66Met Genotype on Serotonin Transporter and Serotonin-1A Receptor Binding

**DOI:** 10.1371/journal.pone.0106810

**Published:** 2014-09-04

**Authors:** Christoph Kraus, Pia Baldinger, Christina Rami-Mark, Gregor Gryglewsky, Georg S. Kranz, Daniela Haeusler, Andreas Hahn, Wolfgang Wadsak, Markus Mitterhauser, Dan Rujescu, Siegfried Kasper, Rupert Lanzenberger

**Affiliations:** 1 Department of Psychiatry and Psychotherapy, Medical University of Vienna, Vienna, Austria; 2 Department of Biomedical Imaging and Image-guided Therapy, Division of Nuclear Medicine Medical University of Vienna, Vienna, Austria; 3 Department of Psychiatry, Medical University of Halle, Halle, Germany; INSERM/CNRS, France

## Abstract

**Background:**

The brain-derived neurotrophic factor (BDNF) Val66Met polymorphism (rs6265) may impact on the *in-vivo* binding of important serotonergic structures such as the serotonin transporter (5-HTT) and the serotonin-1A (5-HT_1A_) receptor. Previous positron emission tomography (PET) studies on the association between Val66Met and 5-HTT and 5-HT_1A_ binding potential (BP_ND_) have demonstrated equivocal results.

**Methods:**

We conducted an imaging genetics study investigating the effect of Val66Met genotype on 5-HTT or 5-HT_1A_ BP_ND_ in 92 subjects. Forty-one subjects (25 healthy subjects and 16 depressive patients) underwent genotyping for Val66Met and PET imaging with the 5-HTT specific radioligand [^11^C]DASB. Additionally, in 51 healthy subjects Val66Met genotypes and 5-HT_1A_ binding with the radioligand [*carbonyl*-^11^C]WAY-100635 were ascertained. Voxel-wise and region of interest-based analyses of variance were used to examine the influence of Val66Met on 5-HTT and 5-HT_1A_ BP_ND_.

**Results:**

No significant differences of 5-HTT nor 5-HT_1A_ BP_ND_ between BDNF Val66Met genotype groups (val/val vs. met-carrier) were detected. There was no interaction between depression and Val66Met genotype status.

**Conclusion:**

In line with previous data, our work confirms an absent effect of BDNF Val66Met on two major serotonergic structures. These results could suggest that altered protein expression associated with genetic variants, might be compensated *in* *vivo* by several levels of unknown feedback mechanisms. In conclusion, Val66Met genotype status is not associated with changes of *in-vivo* binding of 5-HTT and 5-HT_1A_ receptors in human subjects.

## Introduction

The brain-derived neurotrophic factor (BDNF) is the most prominent member in the neurotrophin family and involved in development and activity-dependent regulation of neuronal structures [1]. Cumulating evidence demonstrated a functional interplay between BDNF and the neurotransmitter serotonin (5-HT), constituting common intracellular signaling pathways and transcription factors, BDNF control over the development and function of serotonergic neurons as well as serotonergic regulation of BDNF gene expression and signaling [2].

Briefly, BDNF is linked with at least three major intracellular signaling cascades: the phosphoinositide-3 kinase pathway enabling cell survival, the phospholipase-gamma pathway effecting synaptic plasticity and the mitogen-activated protein kinase pathway associated with neuronal differentiation and neurite outgrowth [3]. Beside the p75 neurotrophin receptor, which is activated by proBDNF and all other neurotrophins, BDNF releases it’s effects by binding to tropomyosin-kinase related receptor B (TrkB) [4]–[6]. Thereby, BDNF is a major factor in the proper development and plastic regulation of the central nervous system and highly active in limbic structures such as the hippocampus and the amygdala, where long-term potentiation, learning and memory are facilitated [7]. However, it should be stated here that most of the evidence of BDNF in this context is based on rodent data.

The BDNF gene is located at chromosome 11p13-14, including many splice sites and promoters. All BDNF mRNAs are initially translated into proBDNF and are then cleaved into mature BDNF [8]. The most investigated polymorphism of the BDNF gene exists in the codon 66 of proBDNF (Val66Met, rs6265) and consists of a valine to methionine substitution, which is associated with reduced intracellular proBDNF trafficking, synaptic secretion of BDNF, and thus a lower extracellular BDNF concentration in met-allele carriers [9]. Thought to trigger deficits in neuronal development and plasticity, the Val66Met polymorphism is of major interest in neuropsychiatric research [2], [7].

Interestingly, in humans the molecular connections between 5-HT and BDNF, and how alterations in one system affect the other are hardly known. Due to the lack of current methods to measure BDNF, TrkB or p75 in the living human brain, *in* *vivo* research in humans mainly focuses on the investigation of alterations of serotonergic structures thought to be mediated via changes in BDNF. In imaging genetics studies, serotonergic markers are labeled by radioligands and their binding is measured using PET. As yet, there exist three studies investigating alterations of BDNF, as represented by the Val66Met polymorphism, and it’s association with binding of 5-HT_1A_, 5-HT_2A_ receptors as well as the 5-HTT in the human brain [10]–[12]. Two previous studies failed to detect links between Val66Met and binding of 5-HT_1A_ and 5-HT_2A_ receptors. On the other side, a recently published study reports lower 5-HT_1A_ binding in healthy subjects carrying the met-allele compared to val-homozygotes, a difference which was not observed in depressed subjects [12]. As far as 5-HTT is concerned, in one study, applying the serotonin transporter (5-HTT) specific radioligand [^11^C]-MADAM (N = 25) with PET and [^123^I]-ß-CIT (N = 18) with single photon emission tomography (SPECT) in two independent samples, the authors found increased 5-HTT binding in val-homozygote male subjects and compared to met-allele carriers [10]. On the other hand applying the radioligand [^11^C]DASB (N = 49), the second study failed to detect any effect of Val66Met genotype status on 5-HTT binding [11].

To resolve contradictory results we conducted an imaging genetics study investigating the association between 5-HTT binding using PET with the radioligand [^11^C]DASB and the Val66Met genotype status in healthy subjects as well as in depressive patients. We also measured 5-HT_1A_ receptor binding in healthy subjects genotyped for Val66Met, in order to resolve two equivocal findings. We hypothesized, that Val66Met impacts on 5-HTT binding in patients with major depression and healthy subjects. Furthermore, we hypothesized that significant differences are detected between BDNF genotype status and 5-HT_1A_ binding in healthy subjects.

## Methods

### Subjects

In a neuroimaging genetics study with a cross-sectional design in total 92 subjects, aged 18–65 years were included. The study was divided into two groups, in the first one 51 healthy adult volunteers (37 female) were included and measured with [*carbonyl*-^11^C]WAY-10063. In the second group 25 healthy subjects (HS) and 16 currently depressed patients with an Hamilton Depression Rating Scale ≥16 (HAMD: 19.7±3.5, mean ± SD) were included (for further details see table 1) and measured with [^11^C]DASB. None of the subjects received both radioligands. The study population originates from a pooled sample, which is part of previously published studies [13]–[16]. Genotyping data of BNDF were previously not published. All subjects underwent a psychiatric screening by the help of the complete Structured Clinical Interview for DSM-IV type disorders (SCID I+II), physical and neurological examination, clinical history, ECG, routine laboratory analysis, urinary drug and pregnancy screening. All subjects were at least three months free of any psychotropic medication. Every study subject was enrolled in study participation after detailed oral information about all study procedures and subsequent signing of a written informed consent form. The study and all study related procedures were approved by the Ethics Committee of the Medical University of Vienna.

**Table 1:** Demographic variables of the entire study sample.

	val/val	met-carrier	p
**healthy subjects**			
** [** ***carbonyl*** **-^11^C]WAY-100635**			
* N ( = 51)*	30	21	
Age (years)	43.8±13.1	45.1±12.36	0.737
Sex (f/m)	21/9	16/5	0.626*
weight	72.9±17.1	67.1±10.5	0.169
SA	296.9±269.1	285.7±197.3	0.702
** [^11^C]DASB**			
* N ( = 25)*	19	6	
Age (years)	31.0±8.8	33.0±13.2	0.672
Sex (f/m)	8/11	1/5	0.258*
weight	76.7±12.1	80.2±10.8	0.537
SA	44.1±47.7	25.6±25.4	0.378
**MDD patients**			
** [^11^C]DASB**			
* N ( = 16)*	13	3	
* HAMD*	19.4±3.6	21±3.5	0.495
* Age (years)*	41.1±8.9	46.7±7.5	0.34
* Sex (f/m)*	9/4	3/0	0.267*
* weight*	77.7±21.3	61.3±2.5	0.251^+^
* SA*	63.9±22.6	62.5±16.7	0.925

Data are given as means ± standard deviations (SD). P-values compare pooled BNDF Val66Met genotype groups with independent sample t-test, chi-square(*) or Mann-Whitney U test (+) where appropriate.

### BDNF Genotyping

All procedures were performed as previously described [13]. Briefly, DNA was isolated from peripheral blood mononuclear cells by the QIAamp DNA Mini-Kit (QIAGEN, Hilden, Germany). Genotyping of BDNF rs6265 single nucleotide polymorphism (SNP) was conducted with the MassARRAY platform (SEQUENOM, San Diego, CA) as described elsewhere [17]. PCR-primers were generated with the Assay Designer 4.0 software (SEQUENOM). Multiplex PCR reactions were performed with 12.5 ng of genomic DNA, 500 µM dNTPs (ABgene, Hamburg, Germany), 100 nM PCR primers, 1.625 mM MgCl2 and 0.5 U HotStar Taq polymerase (QIAGEN). Shrimp alkaline phosphatase (SAP) treatment, an iPLEX reaction cocktail with extension primers (7–14 µM), a iPLEX termination mix and an iPLEX enzyme (SEQUENOM) were added to the PCR-products. The resulting extension products were desalted using SpectroCLEAN resin (SEQUENOM), then spotted on SpectroCHIPs GenII (SEQUENOM) and analyzed with the MassARRAY MALDI-TOF mass spectrometer. Typer 3.4 Software was used to identify allele specific extension products and resulting genotypes (SEQUENOM). For genotyping quality assurance CEU HapMap Trios (Coriell Institute for Medical research, Camden, NJ) were included and compared with the HapMap-CEU population (www.hapmap.org). For all analyses val/val homozygotes ( = GG-carriers) were compared against met-carriers (AG- and AA-carriers).

### Radiochemistry of [^11^C]DASB and [*carbonyl*-^11^C]WAY-100635 and PET Procedures

Radioligand synthesis and all PET measurements were conducted at the Department of Biomedical Imaging und Image-guided Therapy, Division of Nuclear Medicine at the Medical University of Vienna. PET measurements were performed with a GE Advance full ring PET scanner (General Electric Medical Systems, Waukesha, WI, USA). Subjects were placed with their head parallel to the orbitomeatal line guided by a laser beam system to ensure full coverage of the neocortex and the cerebellum in the field of view (FOV). A polyurethane cushion and head straps were used to minimize head movement and to guarantee a soft head rest during the whole scanning period.

For a complete description of [^11^C]DASB radioligand synthesis see [18]. Mean injected dose was 358.97±70.47 MBq, specific activity at time of injection was 49.00±38.10 MBq/nmol and radiochemical purity was above 95%. After a 5 min transmission scan with retractable ^68^Ge rod sources the 3D dynamic emission measurement was initiated simultaneously with the intravenous bolus injection of the radioligand [^11^C]DASB. The total acquisition time (35 slices) was 90 min and reconstructed images comprised a spatial resolution of 4.36 mm full-width at half-maximum (FWHM).

For a complete description of [*carbonyl*-^11^C]WAY-100635 please see [19], [20]. Mean injected dose was 312.04±105.84 MBq, specific activity at time of injection was 285.47±251.22 GBq/µmol and radiochemical purity was above 95%. Again, a 5 min transmission scan (^68^Ge) was followed by 90 min dynamic scanning per subject at a spatial resolution of 4.36 mm FWHM.

### Data preprocessing and calculation of binding potential

PET preprocessing was done in SPM8 (Wellcome Trust Centre for Neuroimaging, London, UK, http://www.fil.ion.ucl.ac.uk/spm/) using standard algorithms and parameters unless stated differently. After realignment to the mean image (quality = 1) scans of the entire time series were summed up and spatially normalized (affine regularization  =  average sized template) to a tracer-specific template within standard MNI-space (Montreal Neurological Institute). Thereafter, the resulting transformation matrix was applied to each time frame.

We assessed in vivo target structure density as indexed by 5-HT_1A_ receptor and 5-HTT binding potentials (BP_ND_), which represent the ratio at equilibrium of specifically bound radioligand to that of nondisplaceable radioligand in tissue [21]. All binding potentials were computed using the voxel-wise modeling tool in the PMOD 3.3 software package (PMOD Technologies, Ltd., Zurich, Switzerland) and applying the two-parameter linearized reference tissue model (MRTM2) [22], which provides advantages in signal-to-noise-ratio, especially for whole-brain voxel-wise analysis.

We modeled 5-HT_1A_ BP_ND_ as previously described by our group using the insula as receptor-rich region and the cerebellum as receptor-poor region [23]. The cerebellar gray matter excluding cerebellar vermis and venous sinus served as reference region. Serotonin transporter BP_ND_ were modeled using the MRTM2 as previously described [16]. In short, k_2_’ was estimated from the striatum as 5-HTT-rich region and the cerebellar gray matter (excl. vermis and venous sinus) as 5-HTT-poor region. The cerebellar gray matter was chosen because it represents an optimal reference region for the quantification of the serotonin transporter with [^11^C]DASB [24], [25]. Regions of interest (ROI) for both radioligands were taken from an automated anatomical labeling-based (AAL) atlas [26] after normalization of BP_ND_ maps to standard MNI-space. Values were averaged across both hemispheres. Due to inherent smoothness of PET data of the scanner and temporary smoothing during normalization we did not smooth during statistical processing.

### Statistical Analysis

For normally distributed demographic variables and clinical measures student’s t-tests, for nominal variables chi-squared tests were performed. Significance was determined as p<0.05 and all tests were two-sided.

Differences of 5-HT_1A_ and 5-HTT BP_ND_ between BDNF Val66Met genotype groups were calculated using a voxel-wise and a ROI-based approach. For the voxel-wise analysis both in the 5-HTT and the 5-HT_1A_ – groups an ANOVA was performed as implemented in SPM8. Grouped genotype status (val/val, vs. met-carrier  =  GG vs. A-carrier) served as factor and radioligand specific activity, sex and age served as covariates. In the 5-HTT-collective diagnosis was added as additional factor in a second step analysis. F-tests and group-wise post-hoc t-tests between genotype groups were calculated and contrasted in SPM8. Additionally, in the 5-HTT-group an interaction between diagnosis and genotype status was contrasted by weighting contrast vectors in SPM according to group size. An absolute image threshold was set at 0.1 BP_ND_ to remove voxels with low signal-to-noise ratio and a cluster threshold was set at 50 voxels. A statistical level of p<0.05 corrected for multiple comparisons by the family-wise error rate (FWE) at voxel-level was considered significant, for subsequent explorative analysis an uncorrected threshold of p<0.001 was accepted.

**Figure 1:**
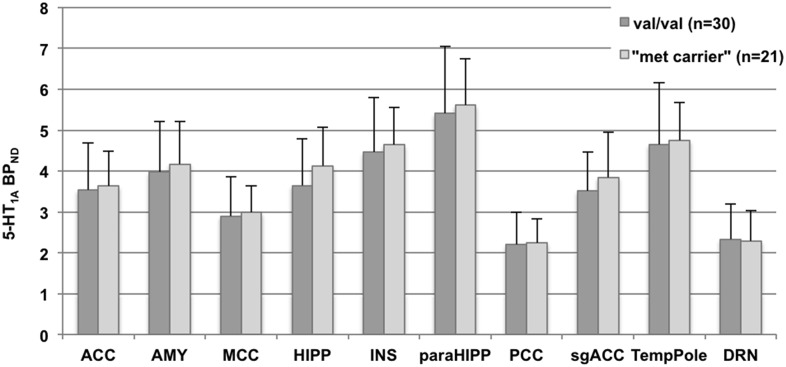
Bar chart plotting serotonin-1A binding potential (5-HT_1A_ BP_ND_) according to BNDF Val66Met genotype status. Values at the y-axis represent 5-HT_1A_ BP_ND_ separated for val/val and met-carrier, respectively, x-axis shows regions of interest. Regions and values correspond to table 2. ACC: anterior cingulate cortex, AMY: amygdala, MCC: medial cingulate cortex, HIPP: hippocampus, INS: insula, paraHIPP: parahippocampus, PCC: posterior cingulate cortex, TempPole: temporal pole, DRN: dorsal raphe nucleus.

**Figure 2:**
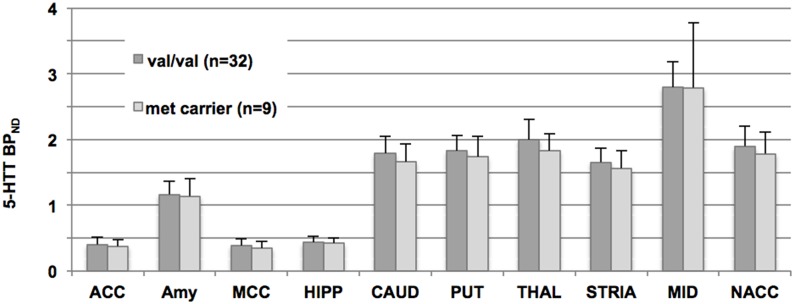
Bar chart plotting serotonin transporter binding potential (5-HTT BP_ND_) according to BNDF Val66Met genotype status. Values at the y-axis represent 5-HTT BP_ND_ in pooled healthy subjects and depressive patients. Binding potential is separated for val/val and met-carriers, respectively, x-axis shows regions of interest. Because healthy subjects and depressive patients were pooled here, regions do, but values do not correspond to table 3. ACC: anterior cingulate cortex, AMY: amygdala, MCC: medial cingulate cortex, HIPP: hippocampus, CAUD: caudatum, PUT: putamen, THAL: thalamus, STRIA: striatum, MID: Midbrain, NACC: nucleus accumbens.

In the ROI-based analyses differences between genotypes groups (val/val vs. met carrier) were calculated with a linear mixed model in SPSS 19 (IBM Corp. Released 2010. IBM SPSS Statistics for Windows, Version 19.0. Armonk, NY: IBM Corp.). Thereby, subject served as the random effect and BDNF genotype status, region, sex and age served as fixed effects. Ten representative regions were chosen due to their a priori known high distribution of 5-HT_1A_ receptors and 5-HTT and implications in psychiatric disorders (see tables 1,2 and figures 1,2). Diagnosis was taken as additional factor in the 5-HTT-study collective. Significance was determined as p<0.05. Post-hoc t-tests were conducted two-sided in 10 AAL ROIs (see tables 1,2 and figures 1,2).

**Table 2:** Post-hoc t-tests comparing serotonin-1A receptor (5-HT_1A_) binding potential (BP_ND_) according to BDNF Val66Met genotype status in 51 healthy subjects.

healthy subjects [*carbonyl*-^11^C]WAY-100635			
region	val/val (n = 30)	met-carrier (n = 21)	p
Anterior cingulate cortex	3.54±1.14	3.63±0.86	0.758
Amygdala	3.98±1.23	4.17±1.03	0.559
Medial cingulate cortex	2.9±0.97	2.98±0.65	0.723
Hippocampus	3.64±1.14	4.12±0.94	0.118
Insula	4.46±1.33	4.64±0.91	0.596
Parahippocampus	5.41±1.64	5.60±1.14	0.596
Posterior cingulate cortex	2.2±0.79	2.25±0.58	0.822
Subgenual anterior cingulate	3.51±0.96	3.85±1.1	0.247
Temporal pole	4.65±1.5	4.75±0.93	0.786
Dorsal raphe nucleus	2.33±0.87	2.29±0.74	0.857

Regions of interest (ROIs) in standardized MNI space (Montreal Neurological Institute) were calculated by automatic anatomical labeling in both hemispheres and averaged. Data are given as 5-HT_1A_ BP_ND_ means ± standard deviations (SD) for each ROI and compared by post-hoc student’s t-tests, values correspond to bar charts in Fig. 1.

## Results

Out of the 51 HS in the 5-HT_1A_-group 30 carried GG, 18 carried AG and 3 AA. The 5-HTT-group had 25 HS with 19 carrying GG, 5 carried AG and 1 AA, whereas in the MDD group with 16 depressed patients 13 carried GG, 3 carried AG and 0 the AA allele (table 1). Allele frequencies of the BDNF gene in all study groups were distributed in accordance with the Hardy-Weinberg equilibrium [5-HT_1A_-group: X^2^ = 0.02, p = 0.891, 5-HTT-group HS: X^2^ = 0.72 p = 0.4, MDD patients X^2^ = 0.17 p = 0.68). The AA and AG+GG study groups did not differ in demographical, clinical measures or radiopharmaceutical measures (table 1). The allelic distribution was not associated with diagnosis in the 5-HTT-group (X^2^ = 0.157, p = 0.692).

In the voxel-wise analysis there was no significant association of BDNF genotype (GG vs. A-carrier) status with 5-HT_1A_ BP_ND_ (F-test: all p>0.05 FWE corr. and all p>0.001 uncorr.). Furthermore, there was no significant association of BDNF genotype (GG vs. A-carrier) with 5-HTT BP_ND_ (F-test: all p>0.05 FWE corr. and all p>0.001 uncorr.). There was no interaction between BDNF genotype status, diagnosis or sex and 5-HTT BP_ND_ (t-test: all p>0.05 FWE corr. and all p>0.001 uncorr).

The mixed model analyses of ROIs in the 5-HT_1A_-group, controlling for potential effects of sex, age and specific radioligand activity, yielded no significant difference of 5-HT_1A_ BP_ND_ in selected ROIs between GG homozygotes and A-allele carriers (F = 0.342, df = 1,45, p = 0.562). In the 5-HTT-group, the mixed model revealed no significant difference between 5-HTT BP_ND_ in the selected ROIs between GG homozygotes and A-allele carriers (F = 0,41, df = 1,33, p = 0.526). There was no interaction between diagnosis and allele in the statistical model (p = 0.989). Post-hoc t-tests and average BP_ND_ values for both study groups are shown in table 2 and table 3, BP_ND_-values of allele groups are displayed in figure 1 and figure 2. Here, in the 5-HTT-group, a significant difference between GG and A-carriers was observed in HS in the midbrain (p = 0.040, uncorr., table 3) as well as between GG in HS and GG in MDD patients (p = 0.034, uncorr.), with BP_ND_ increases in GG-carriers, respectively. All other post-hoc tests (5-HT_1A_: GG vs. A-carrier; 5-HTT HS: GG vs. A-carrier, MDD GG vs. A-carrier, HS vs. MDD GG, HS males GG vs. HS males A-carrier) did not yield significant results (all p>0.05 uncorr.).

**Table 3:** Post-hoc t-tests comparing serotonin transporter (5-HTT) binding potential (BP_ND_) according to BDNF Val66Met genotype status in 25 healthy subjects and 16 depressed patients.

region	healthy subjects			MDD patients		
	val/val(n = 19)	met-carrier(n = 6)	p	val/val(n = 13)	met-carrier(n = 3)	p
Anteriorcingulate	0.42±0.08	0.40±0.06	0.759	0.38±0.14	0.32±0.15	0.517
Amygdala	1.24±0.13	1.14±0.17	0.167	1.06±0.24	1.14±0.46	0.685
Medialcingulate	0.40±0.07	0.37±0.08	0.431	0.37±0.13	0.30±0.12	0.395
Hippocampus	0.46±0.08	0.41±0.08	0.206	0.40±0.10	0.44±0.11	0.525
N. caudatus	1.84±0.21	1.73±0.22	0.305	1.72±0.32	1.50±0.35	0.309
Putamen	1.88±0.18	1.85±0.27	0.756	1.75±0.28	1.50±0.30	0.248
Thalamus	2.07±0.23	1.88±0.11	0.071	1.88±0.37	1.72±0.45	0.527
Striatum	1.70±0.16	1.66±0.22	0.624	1.58±0.25	1.37±0.28	0.231
Midbrain	2.91±0.33	2.58±0.31	0.040	2.62±0.41	3.20±1.80	0.382*
N. accumbens	1.95±0.3	1.82±0.26	0.327	1.82±0.30	1.67±0.46	0.572

Regions of interest (ROIs) in standardized MNI space (Montreal Neurological Institute) were calculated by automatic anatomical labeling in both hemispheres and averaged. Data are given as 5-HTT BP_ND_ means ± standard deviations (SD). T-tests or U-test (*) compare differences between val/val and met-carrier for each ROI.

## Discussion

In a voxel-wise analysis as well as in a ROI-based approach, we did not observe significant differences of 5-HT_1A_-receptor BP_ND_ nor of 5-HTT BP_ND_ according to BDNF genotype status. There was no interaction between MDD diagnosis or sex and 5-HTT BP_ND_. In the midbrain, weak increases of 5-HTT-BP_ND_ in healthy subjects between val-homozygotes and met-carriers were found. Furthermore, weak increases of 5-HTT BP_ND_ were observed in the midbrain in val-homozygote healthy subjects compared to val-homozygote MDD patients. There was no association between allelic distribution and major depression. To sum up, all voxel-wise and ROI-based testing yielded negative results and none of the post-hoc tests survived correction.

Our results are in concordance with a previous PET study applying [^11^C]DASB in 49 healthy subjects, where the authors neither detected differences in 5-HTT binding in relation to BDNF genotype nor a correlation between blood BDNF levels and central 5-HTT binding [11]. Additionally, no effect on 5-HT_2A_ binding was shown in this work. Here, the authors calculated the radiotracer BP_ND_ similar to our study by applying a fully automated reference region model (MRTM2) [22] and an automated ROI-delineation. The only other currently published human PET-study investigating the impact of BDNF polymorphisms on 5-HTT binding reports differences in men and shows no effect of genotype status on 5-HT_1A_ binding [10]. Men homozygous for the val-allele exhibited significantly higher 5-HTT binding in regions such as the hippocampus, insula or dorsal raphe compared to met-carrier, while this effect was absent in women. Furthermore, reductions of 5-HTT binding in met-carrier (n = 3) compared to val-homozygotes (n = 6) in an independent [^123^I]-ß-CIT-study with male suicide attempters were demonstrated, but this reduction was absent when pooled with healthy controls. The authors also used a reference region model with [^11^C]-MADAM, a tracer exhibiting a comparable 5-HTT affinity to [^11^C]DASB [27], the ROIs were manually delineated on individual magnetic resonance images (MRI). Notably, our group previously reported strong correlations of BP_ND_ values between automatically and manually delineated ROIs [23]. The radioligand and the method of ROI generation are on these grounds an unlikely source of variance leading to alternative results. Importantly, in search of arguments for this difference, one must mention that the number of male met-carriers in that collective was low (n = 4), which makes the analysis vulnerable to outliers and hence may increase type-I errors. Likewise, our study exhibits a subgroup with a low subject number and indeed we saw an outlier in the MDD met-carrier group (n = 3) when we plotted the individual BP_ND_ values (data not shown). Hence, our results in depressed patients have to be interpreted with caution. But the fact that both the study by Klein et *al*., which exhibits a large sample size of healthy volunteers, as well as our study did not reproduce higher 5-HTT binding in val-homozygote healthy subjects, rather speaks for an absent effect of BDNF Val66Met on 5-HTT binding.

Apart from this, our study agrees with the data by Henningsson et al., on an absent effect of Val66Met on 5-HT_1A_ receptor binding in healthy subjects [10]. Both studies apply the same radioligand, i.e. [*carbonyl*-^11^C]WAY-100635, exhibit an almost identical number of subjects (n = 53 in Henningsson et al.), and modeled 5-HT_1A_ binding by a reference region model (BP_ND_). These results are in contradiction to a recent finding reporting 5-HT_1A_ reductions in healthy met-allele carriers [12], which is not present in MDD patients. In this study 50 healthy subjects and 50 MDD patients were measured with the radioligand [*carbonyl*-^11^C]WAY-100635, yet 5-HT_1A_ binding was calculated by an arterial input function (BP_F_). Most interestingly, when the authors repeated their analysis with BP_ND_ values, the reduction of 5-HT_1A_ binding in healthy met-carriers was not detectable, suggesting that this finding was associated with the method of radioligand modeling. Following the discussion of the authors, one cannot rule out that Val66Met causes differences of radioligand binding in the blood leading to a bias in the arterial input function. Although, our results are in agreement with all previous studies on 5-HT_1A_ binding using reference tissue models [10], [12], validation by a different tracer not susceptible to modeling methodology is further needed. Taken together, while there are currently contradicting findings on the *in* *vivo* effect of BDNF Val66Met genotypes on 5-HTT binding [10], [11], this study adds data emphasizing the absence of such an effect. Moreover, this work corroborates previous results by reference tissue models demonstrating no association between BDNF Val66Met genotype status and 5-HT_1A_ receptor binding [10], [12] and is in contradiction with a study reporting binding values modeled with arterial blood sampling [12].

Preclinical data report that BDNF promotes development and function of serotonergic neurons by enhancing survival and differentiation [28], increasing local 5-HT [29] modifying the firing pattern of serotonergic raphe neurons [28], [30] and altering the function of serotonergic receptors such as the 5-HT_1A_ and 5-HT_2A_ receptors and the 5-HTT [2], [29], [31]. Vice-versa, raised extracellular 5-HT levels occurring upon administration of SSRIs are thought to increase local BDNF levels by enhanced phosphorylation of serotonergic receptor coupled cAMP response element-binding (CREB) protein [32]–[34], a common target of BDNF and G protein-coupled serotonergic receptors [2]. Confronted with this evidence, one is puzzled upon the lack of strong evidence for an association between BDNF and serotonergic structures in humans *in* *vivo*. However, preclinical studies are not consistent and negative results regarding the expression of 5-HT receptors and transporter are reported [31], [35]. Although the interaction between the BNDF and 5-HT provides a promising bridge between structural and functional neuronal activity, and serves as explanatory hypothesis for neuronal plasticity deficits in neuropsychiatric disorders, exact mechanisms underlying the regulation of the cross connection between BDNF and 5-HT in humans remain unresolved [36]. Our data in concert with above referred work speak for a similar expression of 5-HTT and 5-HT_1A_ receptors upon life-time BDNF reduction, but unfortunately do not illuminate the mechanisms leading to this observation. Theoretically, counter-regulatory or compensatory effects may have altered 5-HTT and 5-HT_1A_ expression. Furthermore, it is possible that not absolute numbers but functional activity of serotonergic structures is altered by BDNF.

The evidence on connections between depression and BNDF genotype status is inconsistent as well. Meta-analytical research suggested an association of Val66Met with major depressive disorder antidepressant treatment response or hippocampal volume and a role of gender and ethnicity [37]–[39]. However, recent meta-analyses refuted these associations and detected power deficits in many trials [40]–[42]. Low serum levels of BDNF were suggested as potential peripheral marker of depression and increase of serum BDNF as response to the appropriate first-line treatment with selective 5-HT reuptake inhibitors (SSRIs). Likewise, this association is weaker than initially thought and there is no relationship between symptom severity and BDNF serum concentration [43]. Our results suggest no association between allelic distribution and diagnosis. Our small number of MDD subjects remain a limiting factor in that regard.

## Limitations

Unfortunately a common problem of human PET studies is weak power resulting from low subject numbers, owed to the large effort of conducting PET-imaging. This is even more intrinsic to genetic PET studies reporting results based on genotype subgroups [44] and in SNP neuroimaging studies where pooling of rare genotype groups is common practice. The low subject number in the MDD met-carrier group could therefore be a limitation of our study. One elegant way to circumvent this issue in future studies would be pooling data between PET centers, which is already common in MRI studies. Second, mean age of genotype groups is heterogeneous, yet controlled for in all statistical analyses. Finally, we did not model PET data with an arterial input function [45], because arterial blood data were not collected. This would have been useful to confirm reported differences according to the methodology for calculating 5-HT_1A_ binding with [*carbonyl*-^11^C]WAY-100635, an issue we are trying to resolve in future studies [46].

## Conclusion

Although others have investigated the effects of the BDNF gene on 5-HTT and 5-HT_1A_ binding with PET, this study adds data to the ongoing discussion about the cross connection between 5-HT and BDNF. While previous work in humans demonstrated contradicting results, due to this work the conclusion of an absent influence of Val66Met on 5-HTT and 5-HT_1A_ has gained substantial support.
