# Correction: Exploring the Impact of BDNF Val66Met Genotype on Serotonin Transporter and Serotonin-1A Receptor Binding

**DOI:** 10.1371/journal.pone.0113173

**Published:** 2014-11-04

**Authors:** The PLOS ONE Staff

The fourth author’s name is spelled incorrectly. The correct name is: Gregor Gryglewski. The correct citation is: Kraus C, Baldinger P, Rami-Mark C, Gryglewski G, Kranz GS, et al. (2014) Exploring the Impact of BDNF Val66Met Genotype on Serotonin Transporter and Serotonin-1A Receptor Binding. PLoS ONE 9(9): e106810. doi:10.1371/journal.pone.0106810
